# Activated Human Mast Cells Induce LOX-1-Specific Scavenger Receptor Expression in Human Monocyte-Derived Macrophages

**DOI:** 10.1371/journal.pone.0108352

**Published:** 2014-09-24

**Authors:** Mervi Alanne-Kinnunen, Jani Lappalainen, Katariina Öörni, Petri T. Kovanen

**Affiliations:** Wihuri Research Institute, Helsinki, Finland; University Heart Center Freiburg, Germany

## Abstract

**Objective:**

Activated mast cells in atherosclerotic lesions degranulate and release bioactive compounds capable of regulating atherogenesis. Here we examined the ability of activated human primary mast cells to regulate the expression of the major scavenger receptors in cultured human primary monocyte-derived macrophages (HMDMs).

**Results:**

Components released by immunologically activated human primary mast cells induced a transient expression of lectin-like oxidized LDL receptor (LOX-1) mRNA in HMDMs, while the expression of two other scavenger receptors, MSR1 and CD36, remained unaffected. The LOX-1-inducing secretory components were identified as histamine, tumor necrosis factor alpha (TNF-α), and transforming growth factor beta (TGF-β1), which exhibited a synergistic effect on LOX-1 mRNA expression. Histamine induced a transient expression of LOX-1 protein. Mast cell –induced increase in LOX-1 expression was not associated with increased uptake of oxidized LDL by the macrophages.

**Conclusions:**

Mast cell-derived histamine, TNF-α, and TGF-β1 act in concert to induce a transient increase in LOX-1 expression in human primary monocyte-derived macrophages. The LOX-1-inducing activity potentially endows mast cells a hitherto unrecognized role in the regulation of innate immune reactions in atherogenesis.

## Introduction

Atherosclerosis is a disease with multifactorial etiology. A complex interplay between dyslipidemia, inflammation, coagulation, fibrinolysis, and endothelial dysfunction is involved in the pathophysiology of the disease. Recent findings in humans and mice suggest that mast cells, previously considered as important mediators of acute allergic reactions, are also multipotent effector cells in atherothrombosis [1]–[4]. In atherosclerotic coronary segments, activated mast cells mediate their effects by releasing histamine, heparin, proteases, prostaglandins, and many cytokines, such as interleukin 1 alpha and beta (IL1-α and β), tumor necrosis factor alpha (TNF-α), transforming growth factor beta-1 (TGF-β1) and interferon gamma (IFN-γ) [5]–[8].

A key event in the formation of atherosclerotic plaques is transformation of macrophages into foam cells. This process is mediated by scavenger receptors (SRs), which enable internalization of modified low-density lipoprotein (LDL) particles, particularly of oxidized LDL (oxLDL) [9]. In mouse peritoneal macrophages, the SRs thrombospondin receptor CD36 and macrophage scavenger receptor MSR1 (aka. SR-A, CD204) account for 78–90% of oxLDL degradation [10]. In human primary macrophages, CD36 accounts for approximately 40% of the oxLDL uptake [11]. In addition to CD36 and MSR1, a third important SR, oxidized low density lipoprotein (lectin-like) receptor 1 (LOX-1, aka. OLR1) has also been shown to promote macrophage foam cell formation [12].

Many pro- and anti-inflammatory mediators released by activated mast cells may influence macrophage SR expression. Previous studies have indicated that, of the mast cell mediators histamine, TNF-α, IFN-γ, IL1-α and β, and TGF-β1 are among potential candidates. Histamine and TNF-α have been reported to induce LOX-1 expression in THP-1 cells [13], [14], and IL1-α and β in smooth muscle cells [15]. The effect of TNF-α on macrophage MSR1 expression is inconsistent, however, one study reported a reduced [16] while another study showed increased [14] MSR1 activity in THP-1 cells upon exposure to TNF-α. In murine J774A.1 macrophages, MSR1 activity is increased in response to TNF-α [17]. Finally, in human primary macrophages, simultaneous treatment with TNF-α and IFN-γ, or with TGF-β1 has resulted in reduced MSR1 and CD36 expression [18], [19].

Based on the above-listed multitude of information on the effects of selected pro- and anti-inflammatory components on SRs in various types of macrophages, we decided to analyze the net effect of the totality of compounds released by activated human primary mast cells (present in the “releasate”) on the expression of the 3 major SRs (MSR1, CD36, and LOX-1) in cultured human primary monocyte-derived macrophages (MDM). We report here that activated mast cells induce LOX-1 expression in human MDM while that of MSR1 and CD36 remains unaffected. This LOX-1-specific effect was synergistically by three individual components released by the activated human mast cells, namely histamine, TNF-α, and TGF-β1.

## Methods

### Reagents and antibodies

Details of antibodies are listed in Table 1 and reagents in Table S1.

**Table 1:** Antibodies used in the study.

Antibody	Catalog no.	Source
Purified human myeloma IgE	DIA HE-1	DiaTec
Polyclonal Goat anti-Human IgE antibody	AP175	Chemicon
Ox-LDL R1 (LOX19-22) antibody	sc-66155	Santa Cruz Biotechnology
CD36 Monoclonal antibody	10009893	Cayman Chemicals
Anti-human macrophage scavenger receptor A monoclonal antibody	Clone SRA-C6	TransGenic
Anti b-actin antibody	8226	Abcam
Polyclonal goat anti-mouse immunoglobulins/HRP	P0447	Dako
Mouse IgA isotype control	ABIN376361	Antibodies Online
IgG1 isotype control	MCA928	AbD Serotech

### Ethics statement

Human plasma and the buffy coats were obtained from healthy blood donors having signed an informed consent. The plasma was a by-product from the preparation of blood products for clinical use. The use of plasma for lipoprotein and cell preparations was approved by the Finnish Red Cross Blood Service (Helsinki, Finland).

### Cell culture

Human monocytes derived from healthy donors were isolated from fresh buffy coats supplied by the Finnish Red Cross Blood Service. Monocytes were differentiated into macrophages in Macrophage SFM medium (Gibco) as previously described [20] with a modification of using M-CSF (50 ng/ml; Nordic Biosite) as the growth factor. Primary human mast cells were differentiated from CD34^+^ progenitor cells according to a protocol developed in our laboratory [21], with a minor modification of using Iscove's Modified Dulbecco's Medium, supplemented with BIT 9500 Serum Substitute (Stemcell Technologies) as the cell culture medium (Mast Cell Culture Medium or MCCM) [22]. Mast cells were grown for at least 8 weeks before the experiments. The LAD2 mast cell line was originally established from a patient with mast cell sarcoma, and kindly provided by Dr. Dean Metcalfe (NIH, USA) [23]. The cells share many characteristics with primary human mast cells and have been commonly used in mast cell research. The LAD2 cells were cultured under serum-free conditions in IMDM containing BIT 9500 serum substitute, L-glutamine (2 mM), 2-mercaptoethanol (0.1 mM), penicillin (100 U/ml), streptomycin (100 µg/ml), and human recombinant SCF (100 ng/ml) [21].

### Activation of mast cells and collection of the releasate

**Figure 1:**
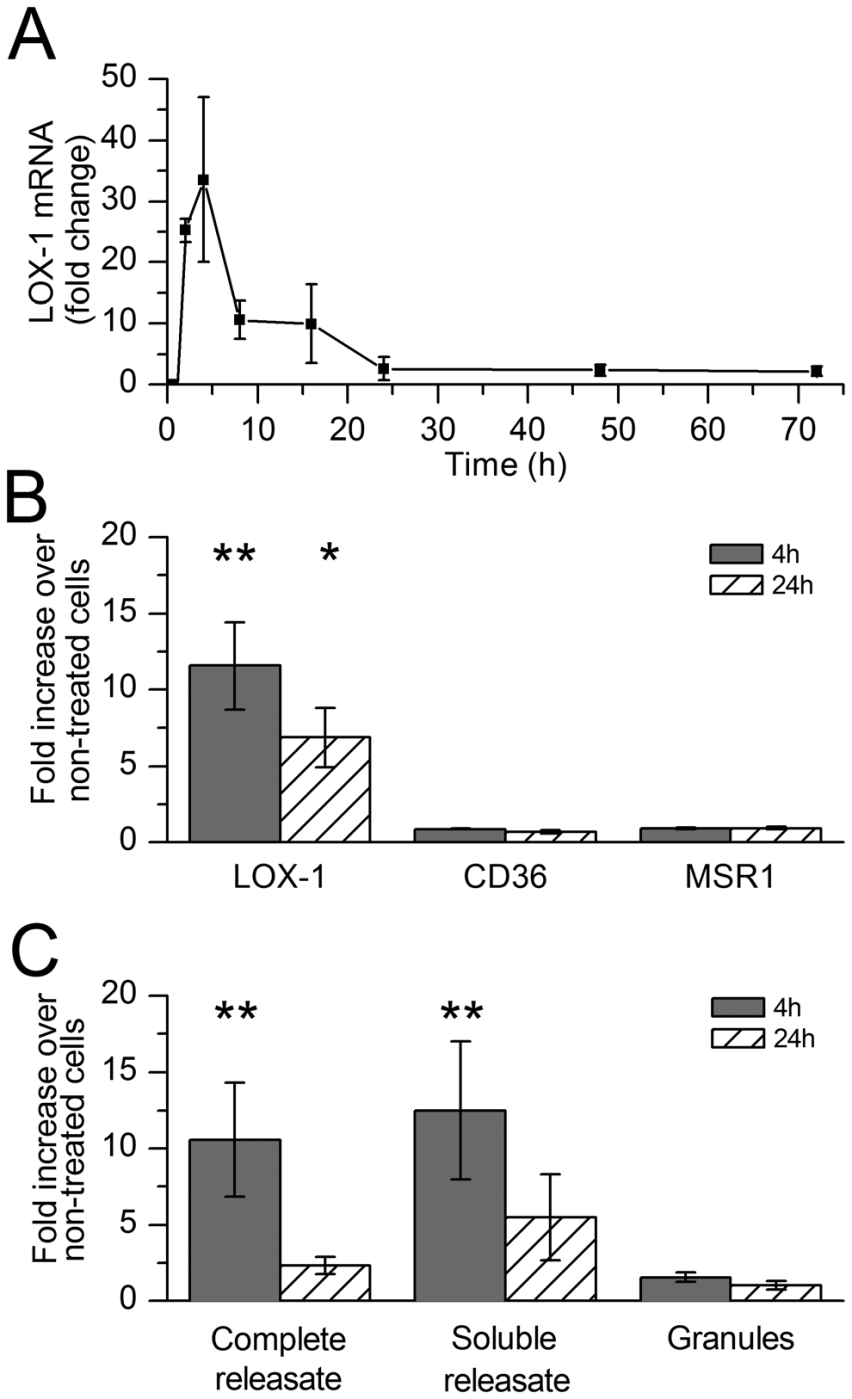
Time-dependent dependent mast cell releasate-induced changes in macrophage scavenger receptor mRNA expression. A) Monocyte-derived macrophages (MDM) from 3 donors were cultured in human primary mast cell releasate for 72 h, and LOX-1 mRNA expression was analyzed and compared to non-treated cells at the beginning of the incubation. B) MDMs from 6 donors were cultured in LAD2 mast cell releasate for 4 h and 24 h, and LOX-1, CD36 and MSR1 mRNA expression was measured and compared to non-treated cells at each time point. C) MDMs from 3 different donors were incubated in complete releasate, soluble releasate, or granules derived from LAD2 mast cells for 4 h and 24 h. The results are presented as means ± SEM. *p<0.05, **p<0.01 vs. non-treated cells.

For immunological activation of mast cells, human primary mast cells and LAD2 mast cells were first sensitized with human IgE (1 µg/ml) for 16 h. After sensitization, the cells were washed and suspended in the Macrophage SFM medium at a density of 1×10^6^ cells/ml (primary cells) or 2×10^6^ cells/ml (LAD2 cells). For the generation of a mast cell releasate, human primary mast cells were initially stimulated to degranulate by incubating them with anti-human IgE (2 µg/ml) in MCCM containing 100 ng/ml of SCF for 30 min (Fig. 1A). Degranulation was confirmed by light microscopy and the presence of histamine in the incubation medium (histamine ELISA, IBL International). To allow addition of the releasate to macrophage cultures in Macrophage SFM, rather than in MCCM, in subsequent experiments the primary mast cells and LAD2 mast cells were stimulated to degranulate in Macrophage SFM, and the medium (releasate) was collected after 1 h incubation. After activation, the cells were sedimented by centrifugation at 200× g for 6 min, the cell supernatant (“complete releasate”) was collected and M-CSF was added to a 50 ng/ml final concentration. Next the granules were recovered by sedimenting them at +4°C, 13000× g for 15 min after which the supernatant (“soluble releasate”) was collected. The granules were washed once with Macrophage SFM medium, centrifuged again as described above and finally suspended in fresh M-CSF-containing Macrophage SFM medium. The complete releasate, the soluble releasate, and the granules were stored at −80°C for further use. In the experiments, a pooled releasate derived from 4 primary mast cell cultures was used.

### Cytokine measurement

The concentrations of TNF-α, IL1-α, IL1-β, and TGF-β1 were measured in the releasates from LAD2 and pooled primary mast cells with Bio-Plex suspension array (Bio-Rad) according to the manufacturer's instructions. Each sample was measured in quadruplicate.

### Macrophage stimulations

Cultured macrophages were washed three times with PBS at room temperature (RT) and incubated with complete releasate, soluble releasate, granules, TNF-α, TGF-β1, IFN-γ, histamine, tryptase, or heparin for the indicated times and concentrations at +37°C. The initial experiment contained approximately 70% of complete releasate and 30% of Macrophage SFM medium, whereas in the subsequent experiments, macrophages were incubated in mast cell releasates prepared in Macrophage SFM medium, as described above.

### Quantitative PCR

Total cellular RNA was extracted from macrophages from duplicate wells by using RNeasy Mini kit with DNase I treatment (Qiagen Inc) and pooled RNA was converted to cDNA with M-MLV reverse transcriptase and random hexamers (Promega). Quantitative real-time PCR technique was performed on an Applied Biosystems' ABI Prism 7500 instrument by using 25 ng of cDNA and either TaqMan or SYBR Green chemistry. The data are expressed relative to glyceraldehyde-3-phosphate dehydrogenase (GAPDH). Detailed description of the oligonucleotide sequences are listed in Table S2 and PCR conditions in File S1.

### Western blot

LOX-1 expression in human primary macrophages was analyzed by Western blot technique using Ox-LDL R-1 (LOX19-22) antibody. Beta-actin was used as a loading control. See Table 1 and File S1 for details.

### Foam cell formation

Human LDL were prepared from plasma of healthy volunteers (Finnish Red Cross Blood Service), as previously described [24]. Native LDL (1 mg/ml) was oxidized at 37°C for 16 h in phosphate buffered saline (PBS with Mg^2+^ and Ca^2+^) containing 10 µg/ml of CuSO_4_. Oxidation was evaluated by measuring the amount of thiobarbituric acid-reactive substances (TBARS). Fluorescent labeling of oxLDL was performed by adding 150 µg of 1,1′-dioctadecyl-3,3,3′,3′-tetramethylindocarbocyanine perchlorate (DiI) in dimethyl sulfoxide to 1 mg (protein) of oxLDL and by incubating the mixture under sterile conditions at 37°C for 16 h. The density of the labeled lipoproteins was raised to 1.064 g/cc with KBr, and the lipoproteins were isolated by ultracentrifugation at 541 000× g with TLA-100.3 rotor for 2 h. Labeled oxLDL was dialyzed 5 times against 500 ml of PBS with 100 µM EDTA and sterile filtered (0.2 µm).

To measure the effect of mast cell complete releasate on macrophage oxLDL uptake, macrophages were treated with DiI-oxLDL (50 µg/ml) and either releasate containing 0.5% BSA or control medium, the composition of which was identical to that of the releasate-containing medium (0.5% BSA, 100 ng/ml of SCF and 2 µg/ml of anti-IgE) for 16 h at 37°C. Before addition of the DiI-labeled oxLDL, macrophages were pre-incubated in releasate or in control medium with neutralizing antibodies against CD36, MSR1, or their isotype controls for 30 min at RT. To assess the extent of non-specific oxLDL binding, macrophages were incubated with 20-fold excess of non-labeled oxLDL for 16 h at 37°C. To evaluate the amount of oxLDL bound to cell surface, the cells were incubated with oxLDL for 1 h on ice. For quantitying oxLDL uptake, macrophages were first washed twice with PBS and twice with 0.5% PBS-bovine serum albumin (BSA), then lysed in 1 g/l of sodium dodecyl sulfate in 0.1 M NaOH, and finally the intensity of DiI fluorescence of the lysate was measured at 544/595 nm with Victor^3^ 1420 Multilabel Counter (Perkin Elmer). Each experiment was performed in duplicate.

### Statistical analysis

The two-sided Mann-Whitney U-test available in the R statistical software package [25] was used for statistical analyses. Statistical significance was set at p<0.05.

## Experimental Results

### Expression of LOX-1 mRNA is induced by releasate derived from LAD2 mast cells

Our initial study with activated human primary mast cells revealed that the products released by them induced LOX-1 mRNA expression in human MDMs. Thus, after addition of the mast cell releasate, LOX-1 expression peaked at 4 h and returned to baseline at 24 h, and remained low thereafter until 72 h (Figure 1A). In subsequent studies, the LAD2 mast cells were utilized and stimulated to degranulate in the macrophage SFM medium, i.e. the macrophage growth medium (see Methods). This enabled us to study the effects of the mast cell-derived products without interference of various cell culture media. The studies clearly showed that the releasate derived from activated LAD2 cells also induced LOX-1 expression, which was 12-fold (p = 0.002) and 7-fold (p = 0.04) over the baseline at 4 h and 24 h, respectively (Figure 1B). Moreover, when studied at 4 h, the effect was found to be dose-dependent (Figure S1). In sharp contrast, the releasate did not influence the expression of CD36 or MSR1 in the human MDMs (Figure 1B). To investigate the individual effect of the exocytosed granule-bound components and the soluble (i.e. not granule-bound) components present in the mast cell releasate on LOX-1 expression, the granules were sedimented by centrifugation, and the experiment was repeated by using the soluble releasate and the granules. We observed that only the complete releasate (11-fold; p = 0.002) and the soluble releasate (13-fold; p = 0.002), but not the granules (1.6-fold; p = 0.3) were able to induce LOX-1 mRNA expression (Figure 1C).

### Characterization of the mast cell-derived releasates

Since the LAD2 mast cell releasate had no effect on the expression of MSR1 and CD36 scavenger receptors, we next identified the components in the mast cell releasates capable of influencing the expression of LOX-1. Previous studies indicate that TNF-α, IL1-α, IL1-β, and TGF-β1 are potent inducers of LOX-1 [14], [15], [19]. In addition, histamine has been shown to increase LOX-1 mRNA expression in THP-1 monocytes, but not in THP-1-derived macrophages [13]. We first characterized the various mast cell releasates with Bio-Plex multiplex suspension array system. The results indicated that the releasate collected from activated LAD2 mast cells and the pooled releasate collected from activated primary mast cells (cultures derived from 4 donors) contained the following concentrations of components, respectively: histamine 0.7 and 7.4 µM; TNF-α 0 and 20 pg/ml; TGF-β1 116 and 87 pg/ml; of IL1-α 3.7 and 0 pg/ml. Neither mast cell releasate contained detectable amounts of IL1-β.

### Histamine, TNF-α, and TGF-β1 induce LOX-1 expression

**Figure 2:**
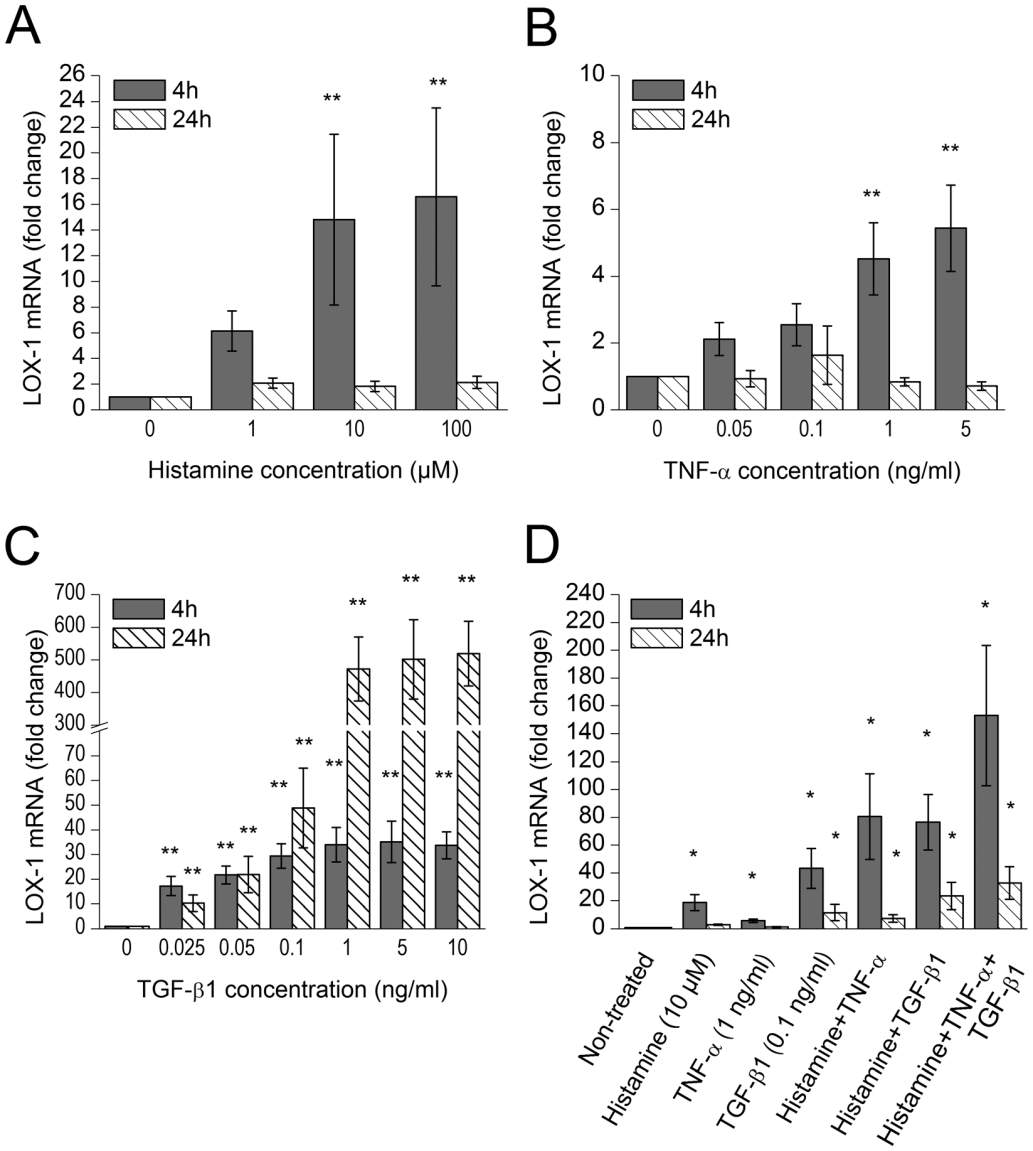
Effect of individual mast cell releasate components on macrophage LOX-1 expression. Monocyte-derived macrophages were incubated with A) histamine, B) TNF-α, and C) TGF-β1 for 4 h and 24 h, and analyzed for LOX-1 mRNA expression (n = 6 donors). D) The combined effect of the components was studied by incubating macrophages with histamine (10 µM) and/or TNF-α (1 ng/ml) and/or TGF-β1 (0.1 ng/ml) for 4 h and 24 h (n = 4 donors). The results are presented as means ± SEM. *p<0.05, **p<0.01 vs. non-treated cells.

**Figure 3:**
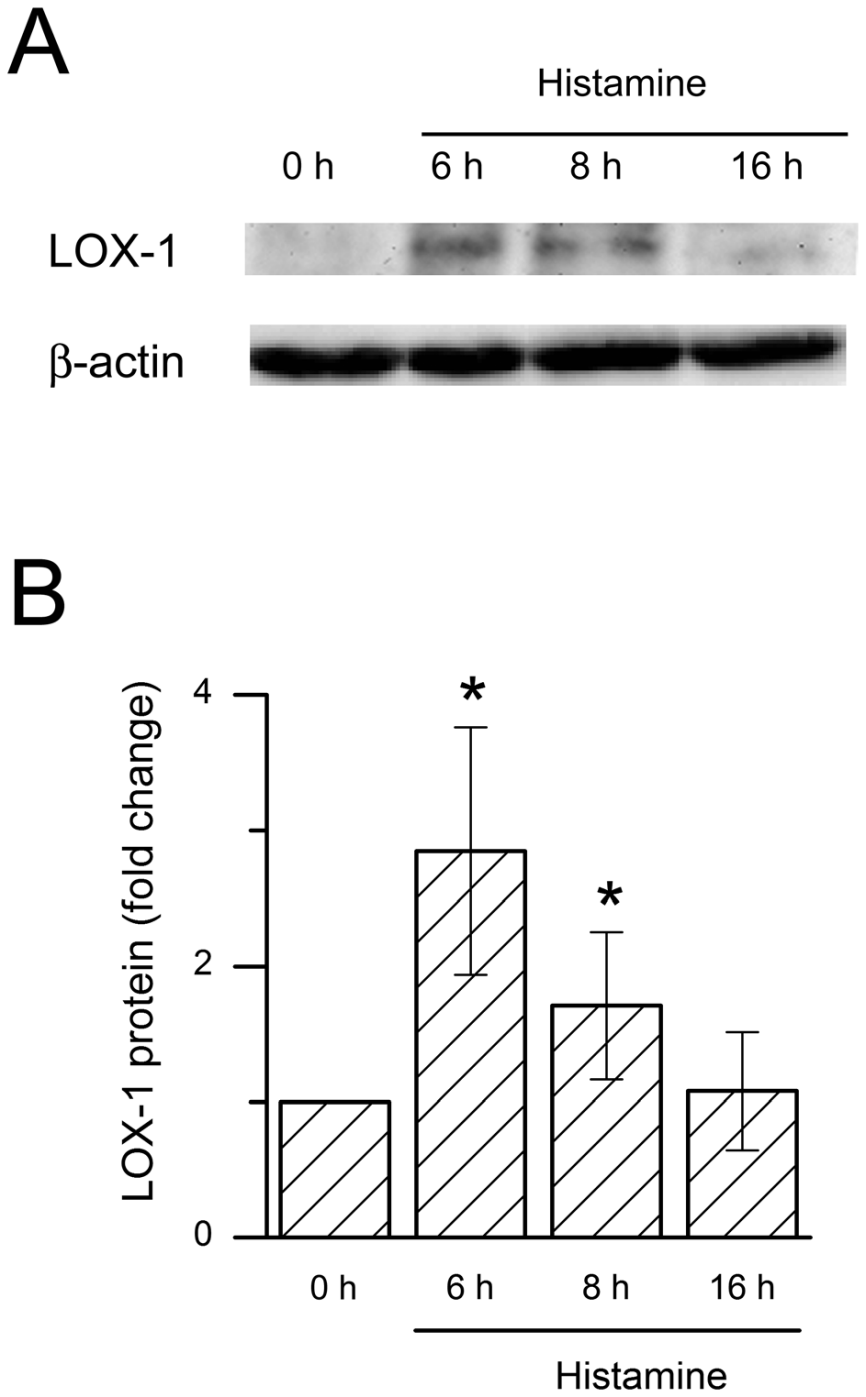
Time-dependent expression of LOX-1 protein in histamine-treated human macrophages. A) Monocyte-derived macrophages were cultured in the presence of 10 µM histamine for 6, 8, and 16 h. The cells were then lysed and LOX-1 protein expression was analyzed by Western blot technique. B) LOX-1 protein levels were quantified, normalized to b-actin, and compared to the levels at the beginning of the incubation (controls). The results are presented as means ± SEM (n = 4 donors). *p<0.05 vs. control cells.

In an attempt to define the components present in the mast cell releasate responsible for the observed LOX-1 induction in human primary MDM, we next incubated human MDMs with histamine, TNF-α, TGF-β1 (all of which were found in the mast cell releasates, see above), and heparin, tryptase and IFN-γ which are characteristic components of any mast cell releasate. We found that histamine, TNF-α and TGF-β1 significantly induced LOX-1 mRNA expression (Figure 2, A–C). Notably, they showed a significant synergistic effect on LOX-1 mRNA expression (Figure 2D). In contrast, heparin, tryptase and IFN-γ failed to induce LOX-1 expression (Figure S2). Next, the macrophages were incubated in the presence of histamine at a concentration (10 µM) similar to what was observed in the primary mast cell releasate for 6 h to 16 h and followed LOX-1 expression at the protein level. We found that the expression peaked at 6 h (Figure 3), which well agreed with the early mRNA response of LOX-1 in the presence of histamine or mast cell releasate (see Figures 1A and 2A).

### Histamine induces an increase in LOX-1 expression mediated via histamine H2 receptor

**Figure 4:**
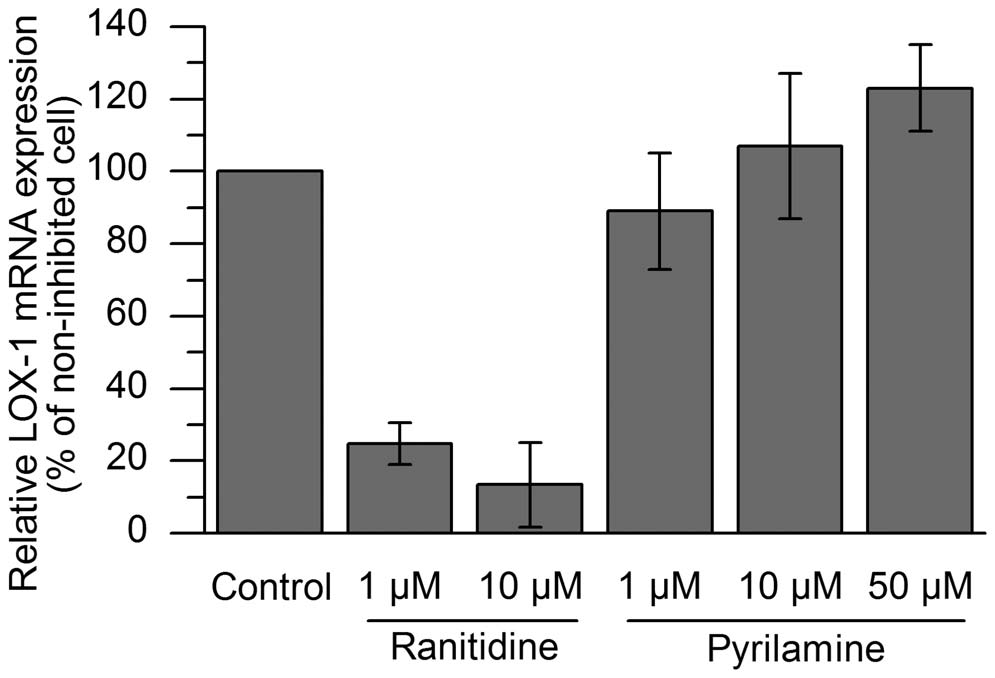
Effect of histamine H1 and H2 receptor antagonists on histamine-induced macrophage LOX-1 mRNA expression. Monocyte-derived macrophages were incubated in the presence of 10 µM histamine and the indicated concentrations of the histamine H1 and H2 receptor antagonists (pyrilamine and ranitidine, respectively) for 4 h and analyzed for LOX-1 mRNA expression. The results are presented as means ± SEM (n = 3 donors). All statistical comparisons were non-significant.

Upon monocyte differentiation into macrophages, the profile of histamine receptors changes. Thus, monocytes primarily express histamine receptor 2 (H2R) whereas macrophages primarily express histamine receptor 1 (H1R) [26], [27]. We measured gene expression of both histamine receptors in monocytes (at 18 h after their isolation from buffy coats), and in differentiated macrophages (at 7 d of culture), and found that these receptors were expressed in monocytes and also in macrophages (Table S3). In the mature macrophages, the histamine-induced LOX-1 mRNA expression was strongly inhibited by ranitidine, an H2R inhibitor, while pyrilamine, an H1R inhibitor, failed to inhibit LOX-1 expression (Figure 4), indicating that the histamine-induced LOX-1 expression was mediated via the H2R.

### Mast cells fail to induce LOX-1-mediated oxLDL uptake by human primary macrophages

**Figure 5:**
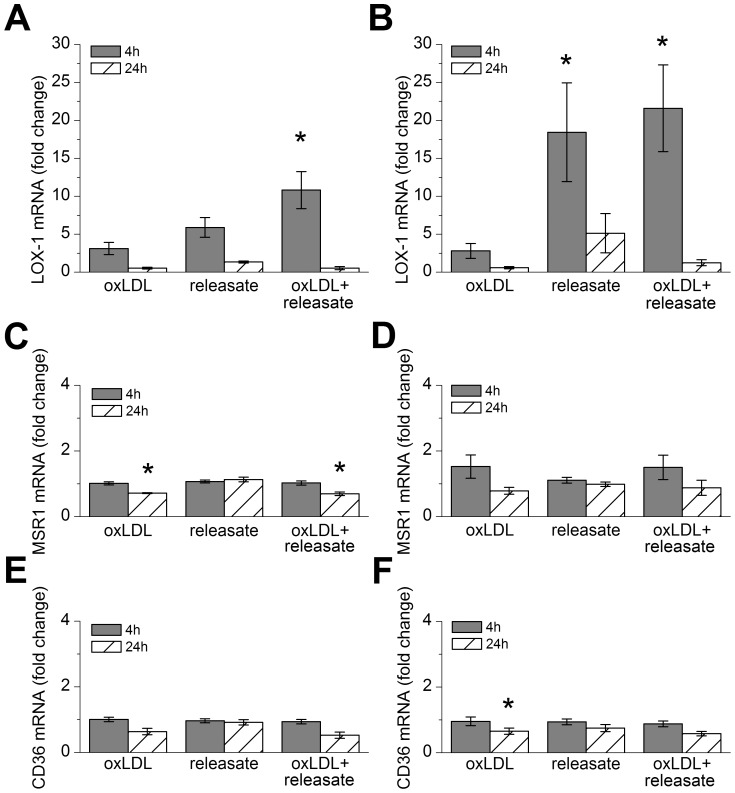
Time-dependent dependent mast cell releasate-induced effects on oxLDL-stimulated macrophage scavenger receptor mRNA expression. Monocyte-derived macrophages were incubated with 50 µg/ml of DiI-oxLDL and/or in LAD2 (A, C, E) or primary mast cell (B, D, F) releasate for 4 h and 24 h, and analyzed for LOX-1, MSR1 and CD36 mRNA expression. The results are presented as means ± SEM (n = 4 donors). *p<0.05; **p<0.01.

**Figure 6:**
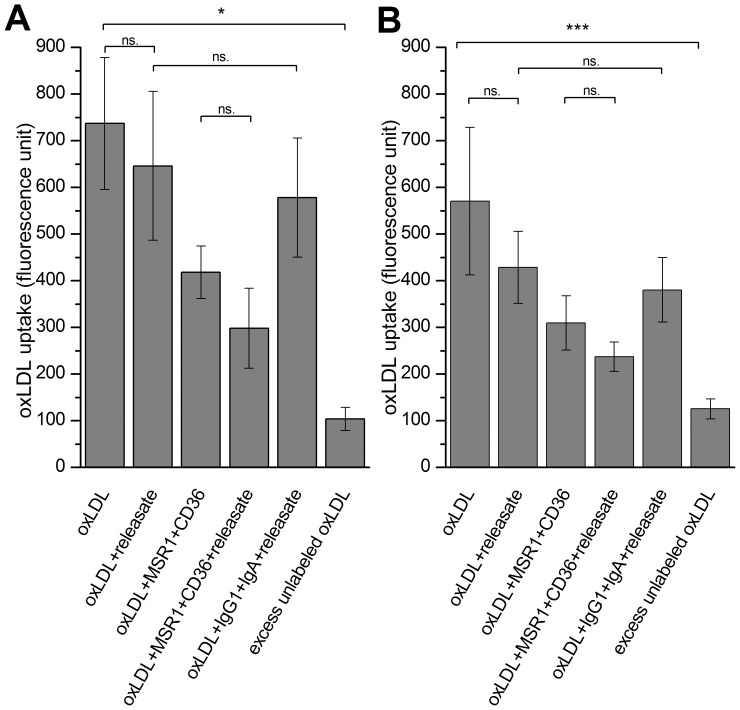
Effect of LAD2 and primary mast cell releasates on macrophage oxLDL uptake. Monocyte-derived macrophages were incubated for 16 h in the presence of 50 µg/ml of DiI-oxLDL and/or with LAD2 (A) or primary mast cell (B) releasate, and/or with specific blocking antibodies for CD36 and MSR1 (2 µg/ml), or with isotype control antibodies IgG1 and IgA (2 µg/ml), or with 20-fold excess of unlabeled oxLDL to determine the magnitude of the high-affinity uptake of Dil-oxLDL. DiI fluorescence was measured from cell lysates. The results are presented as means ± SEM (n = 4 donors). *p<0.05, **p<0.01, ***p<0.001.

We investigated whether the observed transient induction of LOX-1 expression induced by mast cell releasate was sufficient to mediate uptake of oxLDL by the HMDM. For this purpose, macrophages were incubated in the presence of LAD2 mast cell-derived releasate. Moreover, we used specific antibodies to block oxLDL uptake by the 2 major scavenger receptors MSR1 and CD36 in human macrophages. As shown in Figure 5A, in the presence of the releasate LOX-1 mRNA expression was significantly increased, while that of MSR1 and CD36 were not (Figure 5C, E). Incubation of HMDMs for 16 h with DiI-labeled oxLDL (TBARS 35 nmol/mg protein, 20 ng DiI/µg oxLDL) did not result in any detectable increase in the cholesterol content of the cells even in the presence of antibodies that block the uptake of oxLDL by MSR1 and CD36. (Figure 6A) Similarly, the releasate derived from human primary mast cells had no effect on oxLDL uptake in HMDMs (Figure 6B) although LOX-1 mRNA expression was significantly increased and MSR1 and CD36 were unaffected (Figure 5B, D, and F).

## Discussion

In this study, we report that immunologically activated human mast cells release compounds acting as potent inducers of LOX-1 mRNA expression in human primary MDMs, while the expression of two other major macrophage scavenger receptors MSR1 and CD36 remained unaffected. This was attributed to three soluble components released by the activated mast cells, histamine, TNF-α and TGF-β1, which synergistically and significantly increased LOX-1 mRNA expression over a wide range of concentrations.

The results of this study demonstrate that histamine induces LOX-1 mRNA and protein expression in HMDMs via the histamine H2 receptor. Earlier, Tanimoto and colleagues [12] have shown that histamine induces LOX-1 mRNA expression in THP-1 monocytes, but not in THP-1 macrophages. In that study, the lack of the histamine effect on LOX-1 mRNA in THP-1 macrophages was interpreted to depend on the reduction of histamine H2 receptor upon monocyte-to-macrophage differentiation. Although a similar decrease in H2 receptor expression during macrophage differentiation was also observed in the present study, we could, by using specific histamine receptor inhibitors, demonstrate that histamine functions via H2R in HMDMs even when the receptor is expressed at a low level. We also observed that despite of the presence of H2R in primary human monocytes, histamine failed to induce LOX-1 mRNA expression in these cells (data not shown). This observation contrasts the results of Tanimoto et al. and indicates inherent differences, such as the functionality of histamine receptors between monocytic cell lines and human primary cells, especially as a result of monocyte differentiation into macrophages. Indeed, as has been shown based on gene expression profiles, THP-1 macrophages differ drastically from human MDMs differentiated with M-CSF [28]. In fact, although PMA-treated THP-1 monocytes are often regarded as macrophages, they rather resemble an undifferentiated THP-1 monocyte than human primary macrophage. Thus, it is possible that the histamine H2 receptor expression or the downstream signaling molecules of the cAMP pathway activated by this receptor, such as PKA and *creb* vary between THP-1 and HMDM cells.

The observed stimulatory effect of TNF-α and TGF-β1 on LOX-1 expression in HMDMs agrees well with previous publications in which human primary monocytes and THP-1 macrophages have been studied [14], [19]. To our surprise, however, histamine, TNF-α and TGF-β1 synergistically increased LOX-1 expression after 4 h of incubation, suggesting that these components act independently and affect LOX-1 transcription through multiple transcription factors. This is supported by previous reports in which histamine was found to act on LOX-1 via the cAMP-PKA-CRE pathway in THP-1 monocytes [13], and TNF-α to drive LOX-1 expression via the p38 pathway in THP-1 macrophages [14], and TGF-β1 to regulate LOX-1 expression via the NADPH oxidase pathway [29]. However, the combined effect of the three components after 24 h incubation was less than expected from the strong response to TGF-β1, a finding which could be explained by the fact that the strong downregulation of the histamine-mediated induction overrides the effect of TGF-β1.

Although the LAD2 and primary mast cell releasates increased LOX-1 mRNA expression (11-fold and 21-fold, respectively), they did not increase macrophage oxLDL uptake. When macrophages were treated with histamine, we observed that LOX-1 protein expression was only transient: it peaked at 6 h and resumed to almost baseline at 16 h. Presumably, such transient induction was insufficient in increasing LOX-1-mediated oxLDL uptake. Interestingly, also oxLDL has been shown to induce a transient (peak at 6 hours) increase in LOX-1 protein expression in macrophages [30], and TNF-α, IL1-α and IL1-β a transient increase in LOX-1 in smooth muscle cells (peak at 16 hours) [15]. Thus, in contrast to other macrophage SRs, the regulatory machinery determining LOX-1 expression appears to possess elements sensitive to the surrounding stimuli or cholesterol uptake.

In contrast to the present findings in which the combined presence of three LOX-1-upregulating factors released simultaneously from activated mast cells failed to induce any significant uptake of oxLDL, previously Li and colleagues (2004) [12] established delayed and prolonged upregulation of LOX-1 protein expression under high glucose conditions (20 to 30 mmol/L of glucose), which induced oxLDL uptake by human MDMs, and their conversion into foam cells. These data dramatically differ from the rapid and transient mast cell releasate-induced induction of LOX-1 expression observed in the present study.

Scavenger receptors also function as important molecules instigating signaling cascades regulating. macrophage activation, lipid metabolism, pro-inflammatory gene expression, apoptosis, and autophagy [31]. Thus, instead of promoting oxLDL scavenging, mast cell mediators may induce LOX-1 mediated signaling. A recent study has demonstrated that in THP-1 macrophages LOX-1 assumes both pro-inflammatory signaling and scavenging modes upon interaction with oxLDL while facilitating only increased oxLDL scavenging and instigating no signaling events when stimulated simultaneously with IL-10 and oxLDL [30]. It is thus plausible that the role of mast cell induced LOX-1 expression is to launch a signaling cascade and not to increase scavenging of modified LDL particles. Studies in mouse and human macrophages have indicated that LOX-1 mediates expression of pro-inflammatory molecules, such as, ICAM, VCAM, E-selectin and matrix metalloproteinase 3 [32], MCP-1 and TNF-α [33], and facilitates increase in cellular and mitochondrial ROS generation, and expression of NLRP3 inflammasome and autophagy signals [34]. In macrophages, as in endothelial cells [35], the LOX-1 mediated activation of pro-inflammatory gene expression most likely occurs via activation of NADPH oxidase and subsequent intracellular ROS production and NF-kB activation [33]. It has also been shown in endothelial cells that binding of oxLDL to LOX-1 reduces NO availability [36].

## Conclusions

We demonstrate in this study that activated human primary mast cells and LAD2 mast cells induce LOX-1 expression in human primary macrophages *in vitro*. Among the mediators released by the activated mast cells, histamine, TNF-α, and TGF-β1 synergistically induced the expression of LOX-1. It is thus plausible to suggest that *in vivo*-activated mast cells may exert a stimulatory effect on LOX-1 expression in neighboring macrophages, and so exercise a paracrine modulatory effect on macrophage function in tissues in which both types of cell coexist.

## Supporting Information

Figure S1 **Concentration-dependent effect of LAD2 mast cell releasate on macrophage LOX-1 mRNA expression.** Human primary macrophages were incubated with increasing amounts of LAD2 releasate for 4 h and expression of LOX-1 was measured. Mean expression compared to non-treated cells (n = 3) and SEM are presented.
(TIF)

Figure S2 **Effect on macrophage LOX-1 expression of selected mediators contained in mast cell releasate.** Monocyte-derived macrophages were incubated with A) tryptase, B) heparin, or C) interferon gamma for 4 h and 24 h, and analyzed for LOX-1 mRNA expression (n = 4 donors). The results are presented as means ± SEM. All statistical comparisons were non-significant.
(TIF)

Table S1 **Reagents used in this study.**
(DOCX)

Table S2 **qPCR primers and probes (5′–3′ orientation).**
(DOCX)

Table S3 mRNA expression (arbitrary unit*) of histamine receptors 1 and 2 in human monocytes and macrophages from 3 donors.
(DOCX)

File S1 **Detailed methods for quantitative PCR and western blot experiments.**
(DOCX)
