# Supplementary material for: Activated Human Mast Cells Induce LOX-1-Specific Scavenger Receptor Expression in Human Monocyte-Derived Macrophages
Source: PLoS One. 2014 Sep 24;9(9):e108352. doi: 10.1371/journal.pone.0108352 (PMC4176973; doi:10.1371/journal.pone.0108352)
Supplement: Table S1 — Reagents used in this study. (DOCX) [file pone.0108352.s003.docx]

**Table S1: Reagents used in this study.**

| Reagent | Manufacturer |
| --- | --- |
| BIT serum substitute | StemCell Technologies |
| Bovine serum albumin (BSA) | Sigma |
| Clarity Western ECL Substrate | Bio-Rad |
| Histamine (Histamine Dihydrochloride) | Sigma |
| Human recombinant stem cell factor (SCF) | Peprotech |
| Iscove’s Modified Dulbecco’s Medium (IMDM) | Lonza |
| L-glutamine | Lonza |
| Macrophage SFM medium | GIBCO |
| Penicillin-Streptomycin | Lonza |
| Phosphate buffered saline (PBS) | Lonza |
| Polyethylene glycol sorbitan monolaurate (Tween 20) | Fluka |
| Protease inhibitor cocktail, cat# 11873580001 | Roche |
| Pyrilamine | Sigma |
| Ranitidine | Sigma |
| Recombinant human macrophage colony stimulating factor (M-CSF) | Biosite |
| Sodium dodecyl sulphate (SDS) | Sigma |

-------------------------------------------------------------------------------------------------------------------------------------
