# Supplementary material for: Activated Human Mast Cells Induce LOX-1-Specific Scavenger Receptor Expression in Human Monocyte-Derived Macrophages
Source: PLoS One. 2014 Sep 24;9(9):e108352. doi: 10.1371/journal.pone.0108352 (PMC4176973; doi:10.1371/journal.pone.0108352)
Supplement: Table S2 — qPCR primers and probes (5′–3′ orientation). (DOCX) [file pone.0108352.s004.docx]

Table S2. qPCR primers and probes (5’ – 3’ orientation).

| Gene | Forward primer | Reverse primer | Probe | Assay chemistry |
| --- | --- | --- | --- | --- |
| CD36 | gggaaagtcactgcgacatg | tgcaatacctggcttttctca | ttaatggtacagatgcagcctcatttcca | TaqMan |
| GAPDH | ccacatcgctcagacaccat | ggcaacaatatccactttaccagag | ccaatacgaccaaatccgttgactcc | TaqMan |
| LOX-1 | TCGGAAGCTGAATGAGAAATCC | CTTGCGGACAAGGAGCTGA |  | SYBR Green |
| MSR1 | AGGATTTCCAGGTCCAATAGGTC | GAGTCCTCGACTTCCAGGAAAG |  | SYBR Green |

---------------------------------------------------------------------------------------------------------------------------------------------------------------------------------------
