# Supplementary material for: Activated Human Mast Cells Induce LOX-1-Specific Scavenger Receptor Expression in Human Monocyte-Derived Macrophages
Source: PLoS One. 2014 Sep 24;9(9):e108352. doi: 10.1371/journal.pone.0108352 (PMC4176973; doi:10.1371/journal.pone.0108352)
Supplement: Table S3 — mRNA expression (arbitrary unit*) of histamine receptors 1 and 2 in human monocytes and macrophages from 3 donors. (DOCX) [file pone.0108352.s005.docx]

Table S3. mRNA expression (arbitrary unit*) of histamine receptors 1 and 2 in human monocytes and macrophages from 3 donors.

|  | Histamine 1 receptor | | Histamine 2 receptor | |
| --- | --- | --- | --- | --- |
|  | Monocyte | Macrophage | Monocyte | Macrophage |
| Donor 1 | 106465 | 59322 | 7526 | 1288 |
| Donor 2 | 94428 | 79611 | 12684 | 1956 |
| Donor 3 | 113680 | 69633 | 6808 | 3551 |
| Mean±SEM | 104585±5616 | 69522±5857 | 9006±1850 | 2265±671 |

**--------------------------------------------------------------------------------------------**

***** Arbitary unit calculated as 2^((40-average Ct_GAPDH_)-ΔCt)
