# Supplementary material for: Activated Human Mast Cells Induce LOX-1-Specific Scavenger Receptor Expression in Human Monocyte-Derived Macrophages
Source: PLoS One. 2014 Sep 24;9(9):e108352. doi: 10.1371/journal.pone.0108352 (PMC4176973; doi:10.1371/journal.pone.0108352)
Supplement: File S1 — Detailed methods for quantitative PCR and western blot experiments. (DOCX) [file pone.0108352.s006.docx]

# Supporting information S1

## Quantitative PCR

Quantitative PCR was carried out in 25 µl with 1 ng/µl of cDNA in duplicate. Cycling conditions were: initial denaturation step at 95ºC for 10 min, 42 cycles of 95º for 15 s and 60º for 1 min followed by 95º for 15 s, 60 º for 1 min and final denaturation at 95º for 1 min. Assay quality was confirmed by serial dilutions and melt curve analysis, when applicable. Primer3 software was used to design qPCR assays for LOX-1, CD36, MSR1, and GAPDH. LOX-1 assay detects both LOX-1 and LOXIN transcript variants. Primer and probe sequences for are listed in table S2. Histamine receptor-1 and -2 assays were from Qiagen (Hs_HRH1_1_SG, Hs_HRH2_1_SG, respectively). LOX-1 qPCR results are normalized with GAPDH expression. In the initial experiment, the effect of BIT media was eliminated by subtracting 2^(-dCt non-treated cells) from 2^(-dCt releasate treated cells) and then comparing this to the LOX-1 expression at the beginning of the experiment. In subsequent studies, comparative ddCt method was applied and LOX-1 expression was compared to non-treated cells at the same time point.

## Western Blot

Macrophages were lifted from the cell culture dishes by scraping in PBS, pelleted and suspended on ice in RIPA buffer containing 50 mM Tris, 150 mM NaCl, 2 mM EDTA, 1% NP-40, 0.1% SDS, 1x protease inhibitor cocktail. Cell lysis was further facilitated by agitation at +4 ºC and sonication. Protein concentration was determined by BCA assay. 40 µg of total protein lysate was run on 12% SDS-polyacrylamide gel under reducing conditions (100 mM DTT). The proteins were transferred to nitrocellulose membrane (Hybond-C Extra, GE Healthcare Bio-Sciences) with a PerfectBlue Semi-Dry Electro Blotter (Peqlab) at 200mM for 1 h. Non-specific binding was blocked with 5% BSA, 0.075% Tween-20 in 1xTBS buffer (25mM Tris, 140 mM NaCl, 25mM KCl, pH 7.4) at +4 ºC over night after which the blots were incubated with anti-LOX-1 (1:150) and anti-β-actin (1:2000) antibodies at RT for 2 and 1 hours, respectively. Following washing, the blots were incubated for 1 h at RT with anti-mouse immunoglobulin-HRP (1:5000). Membrane-bound antibodies were detected using enhanced chemiluminescence method (Bio-Rad). Protein levels were quantified with a Fiji software.[[35](#_ENREF_35)]
